# Supplementary material for: Unequal power outages induced by natural disasters
Source: Nat Commun. 2025 Oct 8;16:8947. doi: 10.1038/s41467-025-64012-x (PMC12508443; doi:10.1038/s41467-025-64012-x)
Supplement: Supplementary file 1 — Supplementary Information [file 41467_2025_64012_MOESM1_ESM.pdf]

Unequal power outages induced by natural disasters

Bo Wang[1,2], Han Shi[1,2#], Yueming (Lucy) Qiu[3#], Nana Deng[2,4#], Destenie Nock[5], Xingchi Shen[6], Zhaohua Wang[2,4#], Yi Wang[7]

[1]School of Management, Beijing Institute of Technology, Beijing, China.
[2]Digital Economy and Policy Intelligentization Key Laboratory of Ministry of Industry and Information Technology, Beijing, China.
[3]School of Public Policy, University of Maryland College Park, College Park, Maryland, United States.
[4]School of Economics, Beijing Institute of Technology, Beijing, China.
[5]Engineering and Public Policy, Carnegie Mellon University, Pittsburgh, Pennsylvania, United States.
[6]School of International and Public Affairs, Shanghai Jiao Tong University, Shanghai, China.
[7]Department of Economics, Virginia Polytechnic Institute and State University, Blacksburg, Virginia, United States
#Corresponding authors: 15510009875@163.com, yqiu16@umd.edu, dn57160@126.com, wangzhaohua@bit.edu.cn.

Supplementary Table 1. Summary statistics

Varname	Observation	Mean	SD	Min	Max
Natural_disaster	1,504,150	0.0258	0.1593	0	2
Strong_wind	1,504,150	0.0032	0.0562	0	1
Rainstorm	1,504,150	0.0026	0.0513	0	1
Cold_wave	1,504,150	0.0027	0.0523	0	1
Geo_hazard	1,504,150	0.0011	0.0337	0	1
Wildfires	1,504,150	0.0004	0.0199	0	1
Heatwave	1,504,150	0.0157	0.1243	0	1
festival	1,504,150	0.0806	0.2722	0	1
weekend	1,504,150	0.2851	0.4515	0	1

Notes: Natural_disaster represents the intensity of natural disasters, which is the sum of Strong_wind, Rainstorm, Cold_wave, Geo_hazard, Wildfire and Heatwave. Strong_wind is a dummy variable that equals 1 when the county experiences strong winds; Rainstorm is a dummy variable that equals 1 when the county experiences rainstorms; Cold_wave is a dummy variable that equals 1 when the county experiences cold waves; Geo_hazard is a dummy variable that equals 1 when the county experiences geological disasters; Wildfire is a dummy variable that equals 1 when the county experiences wildfire; Heatwave is a dummy variable that equals 1 when the county experiences heatwaves.

Supplementary Table 2. Impacts of grid-related natural disasters on incremental values of power outage frequency

	Outage frequency					
	(1)	(2)	(3)	(4)	(5)	(6)
	ALL	Poverty	Non_Poverty	ALL	Poverty	Non_Poverty
Natural_disaster	0.0031***	0.0051***	0.0028***	0.0028***		
	(0.0003)	(0.0008)	(0.0003)	(0.0003)		
Natural_disaster*Poverty				0.0023***		
				(0.0008)		
Strong_wind					0.0091***	0.0025***
					(0.0023)	(0.0007)
Rainstorm					0.0199***	0.0079***
					(0.0040)	(0.0014)
Cold_wave					0.0070***	0.0019***
					(0.0020)	(0.0005)
Geo_hazard					0.0048**	0.0029**
					(0.0022)	(0.0011)
Wildfire					0.0040	-0.0011
					(0.0035)	(0.0011)
Heatwave					0.0009	0.0020***
					(0.0006)	(0.0003)
Control	YES	YES	YES	YES	YES	YES
County*YM	YES	YES	YES	YES	YES	YES
Observation	1497450	306190	1191260	1497450	306190	1191260
R^2	0.1765	0.1381	0.1914	0.1765	0.1384	0.1915

Notes: * p<0.10, ** p<0.05, *** p<0.01. Standard errors are clustered at the county level and reported below the coefficients. Poverty is a dummy variable, equaling 1 if the county is a poverty county. Control variables include dummy variables for holidays and weekends. The significance of the regression coefficients is assessed using a two-sided t-test, which evaluates whether the coefficients are statistically different from zero. County*YM is the county-month-by-year fixed effect. Observation is the sample size. R2 represents the goodness-of-fit of the regressions.

Supplementary Table 3. Impacts of grid-related natural disasters on incremental values of power outage duration

	Outage duration					
	(1)	(2)	(3)	(4)	(5)	(6)
	ALL	Poverty	Non_Poverty	ALL	Poverty	Non_Poverty
Natural_disaster	0.3257***	0.6929***	0.2569***	0.2565***		
	(0.0452)	(0.1759)	(0.0427)	(0.0427)		
Natural_disaster*Poverty				0.4449**		
				(0.1808)		
Strong_wind					0.2630	0.2478**
					(0.1684)	(0.1184)
Rainstorm					4.0142***	0.8970***
					(1.1589)	(0.2367)
Cold_wave					0.3050**	0.2056***
					(0.1254)	(0.0749)
Geo_hazard					0.6990	0.0885
					(0.4549)	(0.1215)
Wildfire					1.5484	-0.0013
					(1.5854)	(0.2021)
Heatwave					0.2718	0.1486***
					(0.2022)	(0.0444)
Control	YES	YES	YES	YES	YES	YES
County*YM	YES	YES	YES	YES	YES	YES
Observation	1504150	306190	1197960	1504150	306190	1197960
R^2	0.1410	0.1144	0.1550	0.1410	0.1146	0.1550

Notes: * p<0.10, ** p<0.05, *** p<0.01. Standard errors are clustered at the county level and reported below the coefficients. Poverty is a dummy variable, equaling 1 if the county is a poverty county. Control variables include dummy variables for holidays and weekends. The significance of the regression coefficients is assessed using a two-sided t-test, which evaluates whether the coefficients are statistically different from zero. County*YM is the county-month-by-year fixed effect. Observation is the sample size. R^2 represents the goodness-of-fit of the regressions.

Supplementary Table 4. Impacts of grid-related natural disasters on total outage hours

	Total outage hour					
	(1)	(2)	(3)	(4)	(5)	(6)
	ALL	Poverty	Non_Poverty	ALL	Poverty	Non_Poverty
Natural_disaster	0.0494***	0.1230***	0.0358***	0.0356***		
	(0.0074)	(0.0338)	(0.0062)	(0.0062)		
Natural_disaster*Poverty				0.0882**		
				(0.0344)		
Strong_wind					0.0693***	0.0425***
					(0.0258)	(0.0144)
Rainstorm					0.9272***	0.1710***
					(0.2966)	(0.0463)
Cold_wave					0.0587**	0.0120***
					(0.0271)	(0.0044)
Geo_hazard					0.0578	0.0130
					(0.0410)	(0.0099)
Wildfire					0.1653	-0.0153
					(0.1278)	(0.0103)
Heatwave					0.0206	0.0127***
					(0.0159)	(0.0030)
Control	YES	YES	YES	YES	YES	YES
County*YM	YES	YES	YES	YES	YES	YES
Observation	1497450	306190	1191260	1497450	306190	1191260
R^2	0.0868	0.0967	0.0804	0.0868	0.0975	0.0805

Notes: * p<0.10, ** p<0.05, *** p<0.01. Standard errors are clustered at the county level and reported below the coefficients. Poverty is a dummy variable, equaling 1 if the county is a poverty county. Control variables include dummy variables for holidays and weekends. The significance of the regression coefficients is assessed using a two-sided t-test, which evaluates whether the coefficients are statistically different from zero. County*YM is the county-month-by-year fixed effect. Observation is the sample size. R^2 represents the goodness-of-fit of the regressions.

Supplementary Table 5. Robustness check with more control variables.

	(1)	(2)	(3)	(4)
	ln(Outage frequency)	ln(Outage duration)	ln(Outage frequency)	ln(Outage duration)
Natural_disaster	0.0402***	0.0590***		
	(0.0029)	(0.0055)		
Strong_wind			0.0295***	0.0436***
			(0.0058)	(0.0109)
Rainstorm			0.0798***	0.1162***
			(0.0088)	(0.0162)
Cold_wave			0.0376***	0.0660***
			(0.0047)	(0.0095)
Geo_hazard			0.0437***	0.0577***
			(0.0103)	(0.0196)
Wildfire			0.0068	0.0433
			(0.0131)	(0.0329)
Heatwave			0.0359***	0.0496***
			(0.0042)	(0.0081)
Holiday	-0.1614***	-0.3307***	-0.1614***	-0.3308***
	(0.0031)	(0.0051)	(0.0031)	(0.0051)
Weekend	-0.0908***	-0.1678***	-0.0908***	-0.1679***
	(0.0025)	(0.0041)	(0.0025)	(0.0041)
CEPI	0.0178***	0.0147**	0.0178***	0.0148**
	(0.0035)	(0.0071)	(0.0035)	(0.0071)
Coal_price	0.0003***	0.0006***	0.0003***	0.0006***
	(0.0000)	(0.0000)	(0.0000)	(0.0000)
COVID-19	-0.0000***	-0.0000***	-0.0000***	-0.0000***
	(0.0000)	(0.0000)	(0.0000)	(0.0000)
County*YM	YES	YES	YES	YES
Observation	1497450	1504150	1497450	1504150
R^2	0.8412	0.2510	0.8412	0.2510

Notes: * p<0.10, ** p<0.05, *** p<0.01. Standard errors are clustered at the county level and reported below the coefficients. CEPI measures whether there is an environmental inspection team to the province. Coal_price is the price of coal in China per day. COVID-19 measures the number of new infections per city per day. Control variables include dummy variables for holidays and weekends. The significance of the regression coefficients is assessed using a two-sided t-test, which evaluates whether the coefficients are statistically different from zero. County*YM is the county-month-by-year fixed effect. Observation is the sample size. R^2 represents the goodness-of-fit of the regressions.

In this study, our collection of power outage data from utility websites may be subject to sample selection bias, as poverty counties with poor digital infrastructure are systematically more likely to fail to disclose outage information online. The Heckman two-step method, proposed by Nobel laureate James Heckman, is a common and effective solution to addressing the issue of sample selection bias. The Heckman two-step method involves the following steps: First, a selection equation is estimated, which is a probability model for whether an individual is included in the sample. This is typically a Probit model that includes all factors influencing the selection process. Second, an outcome equation is estimated, which examines the relationship between the dependent variable and the explanatory variables. In this step, the inverse Mills ratio (IMR) derived from the first step is incorporated to correct for potential selection bias.

To implement the Heckman two-step model, we require an exclusion restriction variable that affects sample selection but not the outcome variable. This study uses the duration of counties' participation in China's Grassroots Government Information Disclosure Standardization Pilot Program as the exclusion restriction variable. The pilot program, initiated on May 9, 2017, by the General Office of the State Council, designating specific counties as pilot areas, was followed by nationwide implementation on December 26, 2019, to promote the standardization and normalization of grassroots government information disclosure. We code non-participating counties as 0, with values increasing by 1 for each year of participation (1=first year, 2=second year, etc.). Participation in this pilot program significantly increased the probability of power grid-related information disclosure by county governments, as clearly documented in local policy documents. For example, the notice issued by the Jiangbei District People's Government Office in Ningbo City on the comprehensive standardization and normalization of grassroots government information disclosure, as well as the work plan for advancing grassroots government information disclosure standardization and normalization in Yuhang District, both explicitly stipulate the improvement of information disclosure related to the power grid.

Since the Heckman two-step method does not support high-dimensional fixed effects, we first obtained the power outage residuals from a panel regression that controlled for county-month-by-year fixed effects and other control variables using Supplementary Equations(2) and (4). Then, we applied the Heckman two-step method to these power outage residuals. Supplementary Equation(1) is the selection equation, with the duration of participating in the pilot program serving as the exclusive constraint variable. Supplementary Equations(3) and (5) are outcome equations, with the inverse Mills ratio included for regression analysis.

$$\mathrm{pro}\{\mathrm{coll}_{\mathrm{it}}=1\}=\alpha_0+\alpha_1 z_{id}+\alpha_2 \mathrm{Natural\_disaster}_{\mathrm{id}}+\mu_{id} \quad (1)$$

$$\ln(\mathrm{Outage\ frequency}_{\mathrm{id}})=\mathbf{X}'\theta+\delta_{iym}+\varepsilon_{idf} \quad (2)$$

$$\varepsilon_{idf}=\beta_1 \mathrm{Natural\_disaster}_{\mathrm{id}}+\gamma_1 \mathrm{IMR}_{\mathrm{id}}+\varepsilon_{id} \quad (3)$$

$$\ln(\mathrm{Outage\ duration}_{\mathrm{id}})=\mathbf{X}'\theta+\delta_{iym}+\varepsilon_{idd} \quad (4)$$

$$\varepsilon_{idd}=\beta_2 \mathrm{Natural\_disaster}_{\mathrm{id}}+\gamma_2 \mathrm{IMR}_{\mathrm{id}}+\varepsilon_{id} \quad (5)$$

Where $\mathrm{pro}\{\mathrm{coll}_{\mathrm{it}}=1\}$ refers to whether power outage records were collected in this study. The exclusive restriction variable, denoted as z_{id}, represents the duration of participation in the pilot

program. X' represents the remaining control variables. IMR_{id} is obtained from the Supplementary Equation(2) and (4), which can effectively adjust the sample selection bias.

We present the results of addressing the sample selection bias using the Heckman two-step model in Supplementary Table6. We find that natural disasters have a statistically significant and positive impact on power outages (coef = 0.0501***; 0.0847***; Columns 1 and 2, Table S6). The results are consistent with our main results, indicating their robustness. We also examine the unequal power outages caused by natural disasters by introducing interaction terms of natural disasters and the dummy variable of poverty counties (Columns 3 and 4, Table S6). The interaction term "Natural_disaster*Poverty" is significant (coef = 0.0405***; 0.0942***), indicating that the poverty counties experience more outage frequency and longer outage duration caused by natural disasters, showing the robustness of the results.

Supplementary Table 6. The results of the Heckman two-step method

	(1) ln(Outage frequency)	(2) ln(Outage duration)	(3) ln(Outage frequency)	(4) ln(Outage duration)
	1st stage (Selection Model)			
Pilot	0.0591***	0.0587***	0.0581***	0.0577***
	(0.0016)	(0.0016)	(0.0017)	(0.0017)
	2nd stage (Valuation Model)			
Natural_disaster	0.0501***	0.0847***	0.0372***	0.0560***
	(0.0025)	(0.0053)	(0.0021)	(0.0044)
Natural_disaster*Poverty			0.0405***	0.0942***
			(0.0059)	(0.0124)
Control	YES	YES	YES	YES
County*YM	YES	YES	YES	YES

Notes: * p<0.10, ** p<0.05, *** p<0.01. Standard errors are clustered at the county level and reported below the coefficients. Natural disaster represents the intensity of natural disasters. Poverty is a dummy variable, equaling 1 if the county is a poverty county. Pilot represents the duration of participation in the pilot program. The significance of the regression coefficients is assessed using a two-sided t-test, which evaluates whether the coefficients are statistically different from zero. County*YM is the county-month-by-year fixed effect.

Supplementary Table 7. Impacts of grid-related natural disasters on outage frequency across different groups

	ln(Outage frequency)									
	(1)	(2)	(3)	(4)	(5)	(6)	(7)	(8)	(9)	(10)
	Unplanned	Planned	6h-	6h+	North	South	Summer	Winter	0-10	>10
Natural_disaster	0.0350***	0.0104***	0.0220***	0.0254***	0.0108***	0.0552***	0.0452***	0.0191***	0.0165***	0.0443***
	(0.0018)	(0.0023)	(0.0017)	(0.0024)	(0.0033)	(0.0039)	(0.0036)	(0.0032)	(0.0047)	(0.0033)
Control	YES	YES	YES	YES	YES	YES	YES	YES	YES	YES
County*YM	YES	YES	YES	YES	YES	YES	YES	YES	YES	YES
Observation	1497450	1497450	1497450	1497450	738340	759110	753195	744255	644540	852910
R^2	0.9635	0.8628	0.9310	0.8865	0.9012	0.7760	0.8151	0.8688	0.8992	0.7883

Notes: * p<0.10, ** p<0.05, *** p<0.01. Standard errors are clustered at the county level and reported below the coefficients. Control variables include dummy variables for holidays and weekends. The significance of the regression coefficients is assessed using a two-sided t-test, which evaluates whether the coefficients are statistically different from zero. County*YM is the county-month-by-year fixed effect. Observation is the sample size. R2 represents the goodness-of-fit of the regressions. The definition of summer is from May to October. The definition of winter is the remaining months. 0-10 represents counties with fewer than 10 natural disaster occurrences, whereas >10 indicates counties experiencing more than 10 natural disasters during the study period.

Supplementary Table 8. Impacts of grid-related natural disasters on outage duration across different groups

	ln(Outage_duration)									
	(1)	(2)	(3)	(4)	(5)	(6)	(7)	(8)	(9)	(10)
	Unplanned	Planned	6h-	6h+	North	South	Summer	Winter	0-10	>10
Natural_disaster	0.0578***	0.0165***	0.0251***	0.0520***	0.0122*	0.0832***	0.0626***	0.0376***	0.0313***	0.0635***
	(0.0033)	(0.0049)	(0.0024)	(0.0055)	(0.0067)	(0.0075)	(0.0069)	(0.0071)	(0.0095)	(0.0064)
Control	YES	YES	YES	YES	YES	YES	YES	YES	YES	YES
County*YM	YES	YES	YES	YES	YES	YES	YES	YES	YES	YES
Observation	1504150	1504150	1504150	1504150	744370	759780	756565	747585	647890	856260
R^2	0.0980	0.2563	0.2182	0.2321	0.1855	0.2677	0.2598	0.2205	0.2059	0.2615

Notes: * p<0.10, ** p<0.05, *** p<0.01. Standard errors are clustered at the county level and reported below the coefficients. Control variables include dummy variables for holidays and weekends. The significance of the regression coefficients is assessed using a two-sided t-test, which evaluates whether the coefficients are statistically different from zero. County*YM is the county-month-by-year fixed effect. Observation is the sample size. R2 represents the goodness-of-fit of the regressions. The definition of summer is from May to October. The definition of winter is the remaining months. 0-10 represents counties with fewer than 10 natural disaster occurrences, whereas >10 indicates counties experiencing more than 10 natural disasters during the study period.

Supplementary Table 9. Between-group Difference T-test

		T-test		
		Unplanned	Planned	T-statistic(Unplanned-Planned)
Natural_disaster	ln(Outage frequency)	0.0350***	0.0104***	0.0246***
		(0.0018)	(0.0023)	(8.4534)
	ln(Outage duration)	0.0578***	0.0165***	0.0413***
		(0.0033)	(0.0049)	(7.0409)
		6h+	6h-	T-statistic(6h+ - 6h-)
	ln(Outage frequency)	0.0254***	0.0220***	0.0034
		(0.0024)	(0.0017)	(1.1599)
	ln(Outage duration)	0.0520***	0.0251***	0.0269***
		(0.0055)	(0.0024)	(4.4636)

Note: The statistical significance of the T-test is indicated by asterisks: *** $p < 0.01$, ** $p < 0.05$, * $p < 0.10$. All tests were two-sided. The numbers in parentheses in the first two columns represent standard errors. The numbers in parentheses in the last column report the t-values. Adjustments for multiple comparisons were not made.

Supplementary Table 10. Empirical P-Values

Empirical P-Values			
	Variable	Estimate ln(Outage frequency)	Estimate ln(Outage duration)
0-10 versus >10 occurrences	Natural disaster	-0.028^{***}	-0.032^{**}
North versus South		-0.044^{***}	-0.071^{***}
Summer versus Winter		0.026^{***}	0.025^{***}

Notes: We use the empirical p-values to determine if there are significant differences in the coefficients among different groups, which tests for differences between groups using a two-sided test. No adjustments were made for multiple comparisons. Columns 3 and 4 present the results of subtracting the coefficients of natural disasters on power outages in the left-side group from those in the right-side group. We randomly resample 100 times based on bootstrapping to get the empirical p-value across two sub-samples. The null hypothesis is the coefficient estimates for the two sub-samples have no significant difference.

Supplementary Table 11. Robustness check based on interaction term

	(1)<br>ln(Outage frequency)	(2)<br>ln(Outage duration)	(3)<br>ln(Outage frequency)	(4)<br>ln(Outage duration)	(5)<br>ln(Outage frequency)	(6)<br>ln(Outage duration)
Natural_disaster*Poverty	0.0143*	0.0373**				
	(0.0083)	(0.0171)				
Natural_disaster2*Poverty			0.0141*	0.0373**		
			(0.0084)	(0.0171)		
Natural_disaster*Poverty2					0.0172**	0.0300**
					(0.0071)	(0.0142)
Natural_disaster	0.0381***	0.0535***			0.0400***	0.0583***
	(0.0031)	(0.0058)			(0.0038)	(0.0071)
Natural_disaster2			0.0389***	0.0546***		
			(0.0031)	(0.0059)		
Control	YES	YES	YES	YES	YES	YES
County*YM	YES	YES	YES	YES	YES	YES
Observation	1497450	1504150	1497450	1504150	1317220	1321910
R^2	0.8410	0.2505	0.8410	0.2505	0.8306	0.2481

Notes: * p<0.10, ** p<0.05, *** p<0.01. Standard errors are clustered at the county level and reported below the coefficients. Natural_disaster represents the intensity of natural disasters. Natural_disaster2 is an alternative definition of natural disaster, equaling 1 if there are natural disasters on day d in county i. Poverty is a dummy variable, equaling 1 if the county is a poverty county. Poverty2 is an alternative definition of poverty counties, valuing 1 if the county falls in the bottom third of income. Control variables include dummy variables for holidays and weekends. The significance of the regression coefficients is assessed using a two-sided t-test, which evaluates whether the coefficients are statistically different from zero. County*YM is the county-month-by-year fixed effect. Observation is the sample size. R^2 represents the goodness-of-fit of the regressions.

Supplementary Table 12. Impact of grid development on natural-disaster-induced outages.

	(1)<br>ln(Outage frequency)	(2)<br>ln(Outage duration)	(3)<br>ln(Outage frequency)	(4)<br>ln(Outage duration)
Natural_disaster*EGI	-0.1594***	-0.4338***		
	(0.0436)	(0.0784)		
Natural_disaster*LS			-0.1664***	-0.2667***
			(0.0379)	(0.0653)
Natural_disaster	0.0537***	0.0956***	0.0510***	0.0767***
	(0.0049)	(0.0096)	(0.0045)	(0.0086)
Control	YES	YES	YES	YES
County*YM	YES	YES	YES	YES
Observation	1497450	1504150	780550	780550
R^2	0.8410	0.2505	0.8196	0.2331

Notes: * p<0.10, ** p<0.05, *** p<0.01. Standard errors are clustered at the county level and reported below the coefficients. EGI is the cumulative amount of electric grid investment from 2016 to 2019. LS is the labor force in the county's power supply stations. Control variables include dummy variables for holidays and weekends. The significance of the regression coefficients is assessed using a two-sided t-test, which evaluates whether the coefficients are statistically different from zero. County*YM is the county-month-by-year fixed effect. Observation is the sample size. R^2 represents the goodness-of-fit of the regressions.

Supplementary Table 13. Empirical P-Values of poverty and non-poverty counties

Empirical P-Values			
	Variable	Estimate ln(Outage frequency)	Estimate ln(Outage duration)
Poverty versus Non-poverty	Natural_disaster	0.014***	0.036***
	Strong_wind	0.044***	0.039*
	Rainstorm	0.074***	0.139***
	Cold_wave	0.030***	0.053**
	Geo_hazard	0.022	0.076*
	Wildfires	0.003	0.02
	Heatwave	-0.007	0.012
Poverty versus Non-poverty (Unplanned)	Natural_disaster	0.015***	0.036***
Poverty versus Non-poverty (Planned)	Natural_disaster	0.001	0.008
Poverty versus Non-poverty (6h-)	Natural_disaster	0.003	0.008*
Poverty versus Non-poverty (6h+)	Natural_disaster	0.012***	0.031***
Poverty versus Non-poverty (North)	Natural_disaster	0.035***	0.043***
Poverty versus Non-poverty (South)	Natural_disaster	0.007	0.041**
Poverty versus Non-poverty (Summer)	Natural_disaster	0.017**	0.046***
Poverty versus Non-poverty (Winter)	Natural_disaster	0.009	0.012
Poverty versus Non-poverty (0-10)	Natural_disaster	0.046***	0.073***
Poverty versus Non_poverty (>10)	Natural_disaster	0.008	0.029**

Notes: We use the empirical p-values to determine if there are significant differences in the coefficients between poverty and non-poverty counties, which tests for differences between groups using a two-sided test. No adjustments were made for multiple comparisons. Columns 3 and 4 present the results of subtracting the coefficients of natural disasters on power outages in poverty counties from those in non-poverty counties. We randomly resample 100 times based on bootstrapping to get the empirical p-value across two sub-samples. The null hypothesis is that the coefficient estimates for the two sub-samples have no significant difference.

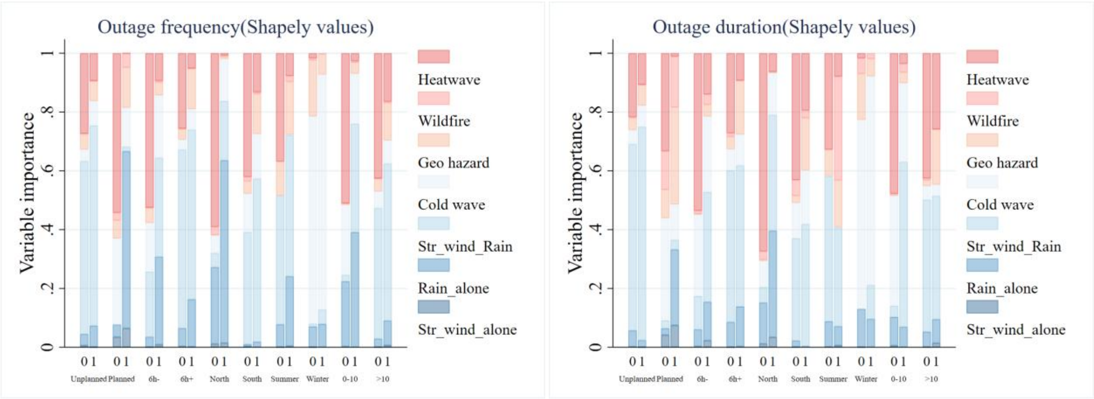

(a) (b)

Supplementary Fig. 1. The contribution decomposition results of natural disaster impact on power outages (considering the isolated impact of rainstorms, strong winds, and their compound occurrence)

Note: In Panels (a) and (b), we show the decomposition results of natural disaster impacts on power outages based on Shapley Values. The 1 and 0 in the X-axis in Panels (a) and (b) represent the poverty and non-poverty counties, respectively. Str_wind_Rain denotes the compound event of simultaneous strong winds and rainstorms. Rain_alone represents the occurrence of rainstorms in isolation, while Str_wind_alone indicates the occurrence of strong winds independently.

Projections under different scenarios

In this section, we estimate future power outages, taking grid infrastructure upgrades into account. We construct three scenarios: (1) the Reference Scenario, where the impact of natural disasters on power outages remains unchanged; (2) the Improvement to Non-Poverty Counties Level Scenario, where the impact of natural disasters on power outages in all poverty-stricken counties is projected to decrease to the current level experienced by non-poverty counties in China by 2060; (3) the Improvement to the Top 20% level Scenario, where the impact of natural disasters on power outages in all counties is projected to reduce to the current level experienced by the top 20% of counties with the highest power investment in China by 2060. In the last two scenarios, considering China's goal to establish a new energy system by 2060 - where China will vigorously improve the comprehensive regulation capability of the power system and accelerate the construction of flexible power regulation to build a strong smart grid and enhance grid security—we assume that counties currently below the target level will gradually reduce the impact of natural disasters on power outages at a steady rate until 2060. After 2060, this impact is expected to stabilize at the target level.

The results are shown in the Table S14. The results indicate that the improvement of emergency response capabilities and grid infrastructure significantly reduces the increase of outage hours caused by changes in natural disasters compared to the Reference Scenarios. Specifically, in SSP-RCP126, the change of natural disasters will increase the outage hours by 0.072 h/person/year in Reference Scenarios, while 0.012 h/person/year in Improvement to the Non-poverty Counties Level Scenario and 0.003 h/person/year in Improvement to the Top 20% Level Scenario. In SSP-RCP585, the change of natural disasters will increase the outage hours by 0.142 h/person/year in Reference Scenarios, compared to 0.077 h/person/year in Improvement to the Non-poverty Counties Level Scenario and 0.063 h/person/year in Improvement to the Top 20% Level Scenario. Furthermore, the gaps between poverty and non-poverty counties in the Improvement to the non-poverty level Scenario and the Improvement to the top 20% level Scenario are also reduced in SSP-RCP126 (-54.33% and -53.90%) and SSP-RCP585 (-67.78% and -66.65%) compared to the Reference Scenarios. These findings suggest that the enhancements in emergency response capabilities and grid infrastructure are significantly important in reducing natural disaster-induced outages and reducing the gaps between poverty and non-poverty counties.

Supplementary Table 14. Impacts of future natural disasters on outage hours in three scenarios

	SSP-RCP126		SSP-RCP585	
	ΔOutage hours (h/person/year)	ΔGaps	ΔOutage hours (h/person/year)	ΔGaps
Reference	0.072	157.82%	0.142	157.76%
Improvement to Non-Poverty Counties LevelLevel	0.012	-54.33%	0.077	-67.78%
Improvement to the Top 20% level	0.003	-53.90%	0.063	-66.65%
