# Supplementary material for: Unequal power outages induced by natural disasters
Source: Nat Commun. 2025 Oct 8;16:8947. doi: 10.1038/s41467-025-64012-x (PMC12508443; doi:10.1038/s41467-025-64012-x)
Supplement: Supplementary file 2 — Description of Additional Supplementary Files [file 41467_2025_64012_MOESM2_ESM.pdf]

**Description of Additional Supplementary Files**

File Name: Supplementary Data 1

Description: Supplementary Data 1 is the source data for Table 1. The table shows the impacts of grid-related natural disasters on power outages. The statistical significance of the coefficients is indicated by asterisks: * denotes $p<0.10$, ** denotes $p<0.05$, and *** denotes $p<0.01$. Standard errors are clustered at the county level and reported below the coefficients. ln(Outage frequency) refers to the natural logarithm of power outage frequency. ln(Outage duration) refers to the natural logarithm of power outage duration. Natural_disaster represents the intensity of natural disasters, which is the sum of Strong_wind, Rainstorm, Cold_wave, Geo_hazard, Wildfire and Heatwave. Strong_wind is a dummy variable that equals 1 when the county experiences strong winds; Rainstorm is a dummy variable that equals 1 when the county experiences rainstorms; Cold_wave is a dummy variable that equals 1 when the county experiences cold waves; Geo_hazard is a dummy variable that equals 1 when the county experiences geological disasters; Wildfire is a dummy variable that equals 1 when the county experiences wildfire; Heatwave is a dummy variable that equals 1 when the county experiences heatwaves. Controls include dummy variables for holidays and weekends. County*YM is the county-month-by-year fixed effect. Observation refers to the sample size. R2 indicates the goodness-of-fit of the regressions. The significance of the regression coefficients is assessed using a two-sided t-test, which evaluates whether the coefficients are statistically different from zero.

File Name: Supplementary Data 2

Description: Supplementary Data 2 is the source data for Panel (a) of Figure 2. The table shows the impacts of Natural disaster, Strong wind, Rainstorm, Cold wave, Geo hazard, Wildfire and Heatwave on power outage frequency. The statistical significance of the coefficients is indicated by asterisks: * denotes $p<0.10$, ** denotes $p<0.05$, and *** denotes $p<0.01$.

File Name: Supplementary Data 3

Description: Supplementary Data 3 is the source data for Panel (b) of Figure 2. The table shows the impacts of Natural disaster, Strong wind, Rainstorm, Cold wave, Geo hazard, Wildfire and Heatwave on power outage duration. The statistical significance of the coefficients is indicated by asterisks: * denotes $p<0.10$, ** denotes $p<0.05$, and *** denotes $p<0.01$.

File Name: Supplementary Data 4

Description: Supplementary Data 4 is the source data for Panel (c) of Figure 2. The table shows the heterogeneity impact of Natural disaster on power outage frequency. The term “group” refers to different heterogeneity grouping characteristics, while “parm” denotes the core explanatory variable of interest. The primary explanatory

variable in this table is “Natural_disaster”, which represents natural disasters. The term "estimate" signifies the estimated coefficient, “stderr” stands for the standard error of the estimate, “t” represents the t-value of the estimated coefficient, “p” indicates the p-value of the estimated coefficient, “min95” denotes the lower bound of the 95% confidence interval of the estimate, and “max95” represents the upper bound of the 95% confidence interval. The term “poverty” is an indicator for county poverty status, where a value of 1 signifies a poverty county, and a value of 0 indicates a non-poverty county. The term “Unplanned” refers to unplanned power outages, whereas “Planned” refers to scheduled power outages. The category “6h-” represents power outages lasting less than 6 hours, and “6h+” represents power outages lasting 6 hours or longer. The definition of “North” pertains to the northern regions of China, and South pertains to the southern regions. The definition of “Summer” is from May to October. The definition of winter is the remaining months. “0-10” represents counties with fewer than 10 natural disaster occurrences, whereas “>10” indicates counties experiencing more than 10 natural disasters during the study period.

File Name: Supplementary Data 5
Description: Supplementary Data 5 is the source data for Panel (d) of Figure 2. The table shows the heterogeneity impact of Natural disaster on power outage duration. The term “group” refers to different heterogeneity grouping characteristics, while “parm” denotes the core explanatory variable of interest. The primary explanatory variable in this table is “Natural_disaster”, which represents natural disasters. The term "estimate" signifies the estimated coefficient, “stderr” stands for the standard error of the estimate, “t” represents the t-value of the estimated coefficient, “p” indicates the p-value of the estimated coefficient, “min95” denotes the lower bound of the 95% confidence interval of the estimate, and “max95” represents the upper bound of the 95% confidence interval. The term “poverty” is an indicator for county poverty status, where a value of 1 signifies a poverty county, and a value of 0 indicates a non-poverty county. The term “Unplanned” refers to unplanned power outages, whereas “Planned” refers to scheduled power outages. The category “6h-” represents power outages lasting less than 6 hours, and “6h+” represents power outages lasting 6 hours or longer. The definition of “North” pertains to the northern regions of China, and South pertains to the southern regions. The definition of “Summer” is from May to October. The definition of winter is the remaining months. “0-10” represents counties with fewer than 10 natural disaster occurrences, whereas “>10” indicates counties experiencing more than 10 natural disasters during the study period.

File Name: Supplementary Data 6
Description: Supplementary Data 6 is the source data for Panel (e) of Figure 2. The table shows the decomposition results of natural disaster impacts on power outage frequency based on Shapley Values. The term “poverty” is an indicator for county poverty status, where a value of 1 signifies a poverty county, and a value of 0 indicates

a non-poverty county. The term “Unplanned” refers to unplanned power outages, whereas “Planned” refers to scheduled power outages. The category “6h-” represents power outages lasting less than 6 hours, and “6h+” represents power outages lasting 6 hours or longer. The definition of “North” pertains to the northern regions of China, and South pertains to the southern regions. The definition of “Summer” is from May to October. The definition of winter is the remaining months. “0-10” represents counties with fewer than 10 natural disaster occurrences, whereas “>10” indicates counties experiencing more than 10 natural disasters during the study period.

File Name: Supplementary Data 7
Description: Supplementary Data 7 is the source data for Panel (f) of Figure 2. The table shows the decomposition results of natural disaster impacts on power outage duration based on Shapley Values. The term “poverty” is an indicator for county poverty status, where a value of 1 signifies a poverty county, and a value of 0 indicates a non-poverty county. The term “Unplanned” refers to unplanned power outages, whereas “Planned” refers to scheduled power outages. The category “6h-” represents power outages lasting less than 6 hours, and “6h+” represents power outages lasting 6 hours or longer. The definition of “North” pertains to the northern regions of China, and South pertains to the southern regions. The definition of “Summer” is from May to October. The definition of winter is the remaining months. “0-10” represents counties with fewer than 10 natural disaster occurrences, whereas “>10” indicates counties experiencing more than 10 natural disasters during the study period.
